# Microwave-Driven Exsolution of Ni Nanoparticles in A-Site Deficient Perovskites

**DOI:** 10.1021/acsnano.3c08534

**Published:** 2023-11-17

**Authors:** Andrés López-García, Aitor Domínguez-Saldaña, Alfonso J. Carrillo, Laura Navarrete, Maria I. Valls, Beatriz García-Baños, Pedro J. Plaza-Gonzalez, José Manuel Catala-Civera, José Manuel Serra

**Affiliations:** † Instituto de Tecnología Química, Universitat Politècnica de València-Consejo Superior de Investigaciones Científicas, Av. dels Tarongers, 46022 València, Spain; ‡ Instituto ITACA, Universitat Politècnica de València, Camí de Vera, 46022 València, Spain

**Keywords:** exsolution, microwave, perovskite, nanoparticle nucleation, hydrogenation, nickel

## Abstract

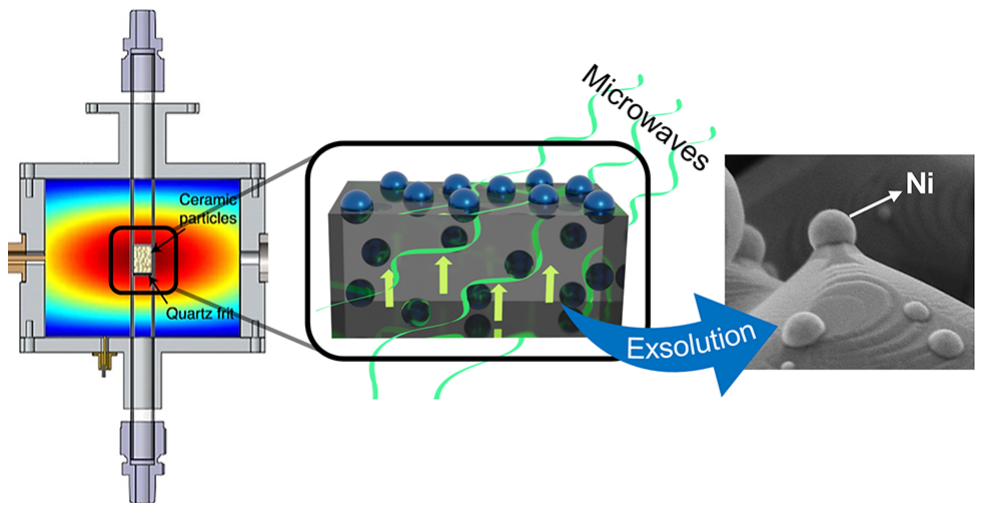

Exsolution has emerged as a promising method for generating metallic nanoparticles, whose robustness and stability outperform those of more conventional deposition methods, such as impregnation. In general, exsolution involves the migration of transition metal cations, typically perovskites, under reducing conditions, leading to the nucleation of well-anchored metallic nanoparticles on the oxide surface with particular properties. There is growing interest in exploring alternative methods for exsolution that do not rely on high-temperature reduction via hydrogen. For example, utilizing electrochemical potentials or plasma technologies has shown promising results in terms of faster exsolution, leading to better dispersion of nanoparticles under milder conditions. To avoid limitations in scaling up exhibited by electrochemical cells and plasma-generation devices, we proposed a method based on pulsed microwave (MW) radiation to drive the exsolution of metallic nanoparticles. Here, we demonstrate the H_2_-free MW-driven exsolution of Ni nanoparticles from lanthanum strontium titanates, characterizing the mechanism that provides control over nanoparticle size and dispersion and enhanced catalytic activity and stability for CO_2_ hydrogenation. The presented method will enable the production of metallic nanoparticles with a high potential for scalability, requiring short exposure times and low temperatures.

## Introduction

Nanostructured materials development has been key to improving crucial catalytic processes in our society. Due to their properties, nanocatalysts proved to be useful for heterogeneous catalysis applications in a wide variety of reactions^1,2^ or as electrocatalysts for energy conversion/storage devices.^3,4^ Metal oxides, such as perovskites (ABO_3_), are widely studied and used for those and other reactions due to their excellent chemical and physical properties and functional versatility.^5,6^ In this context, significant efforts have been made to improve the functionalization of materials with NPs, and exsolution emerges as a promising method for this purpose. Exsolution provides a one-step *in situ* pathway to generate well dispersed, homogeneously sized metallic NPs over the surface of metal oxides^4,7−9^ like cerias^10,11^ (CeO_2_), double perovskites^12−14^ (A_2_B_2_O_6_), or the formerly mentioned perovskites.^15−17^ This method is based on introducing the catalytic active metal (or metals) in the lattice of the metal oxide host when synthesized; these metallic cations will migrate to the material’s surface when subjected to reducing (physical or chemical) agents at relatively high temperatures. There, these metallic cations will form anchored nanoparticles, which give them increased resistance to sintering or coke formation,^18^ among other advantages, like the possibility of reversible exsolution.^19,20^

Due to its many benefits, exsolution is widely studied as an alternative to other functionalization methods, like impregnation. Despite that and following the actual trend, significant efforts are being made to improve the efficiency of exsolution. Especially when referring to the requirements of the treatment: exsolution usually demands, as stated before, medium-to-high temperatures (500–1000 °C) but also relatively prolonged operation times (several hours) and the use of a reducing agent, typically H_2_ flows, constituting the usual thermal exsolution. But these strong reducing conditions are susceptible to be improved. One of the strategies used to achieve this relies on the design of the metal oxide material: A-site deficiency has been proved to be a key factor in the enhancement of exsolution,^21,22^ and thus it can positively impact the exsolution requirements. This has already been stated by Guo et al. in their work, in which a 350 °C exsolution is achieved using A-site deficient double perovskites.^21^ Another possibility is to find alternative methods to thermal exsolution. In this context, two main alternatives have been explored: first, electrochemical poling, developed by Myung et al., which could trigger short-time exsolution, with high dispersion of the formed nanoparticles and in the absence of H_2_ atmosphere.^22^ Despite its many advantages, this method is limited to electrochemical applications due to the need to have the metal oxide deposited and consolidated as an electrode, and it also requires high sintering temperatures. Second, and more recently, plasma-driven exsolution showed improved exsolution compared to the thermal one based on shorter times and lower temperatures required, also with no need for H_2_ supply. Nevertheless, this method requires very low pressure to generate the plasmas.^23,24^

**Figure 1:**
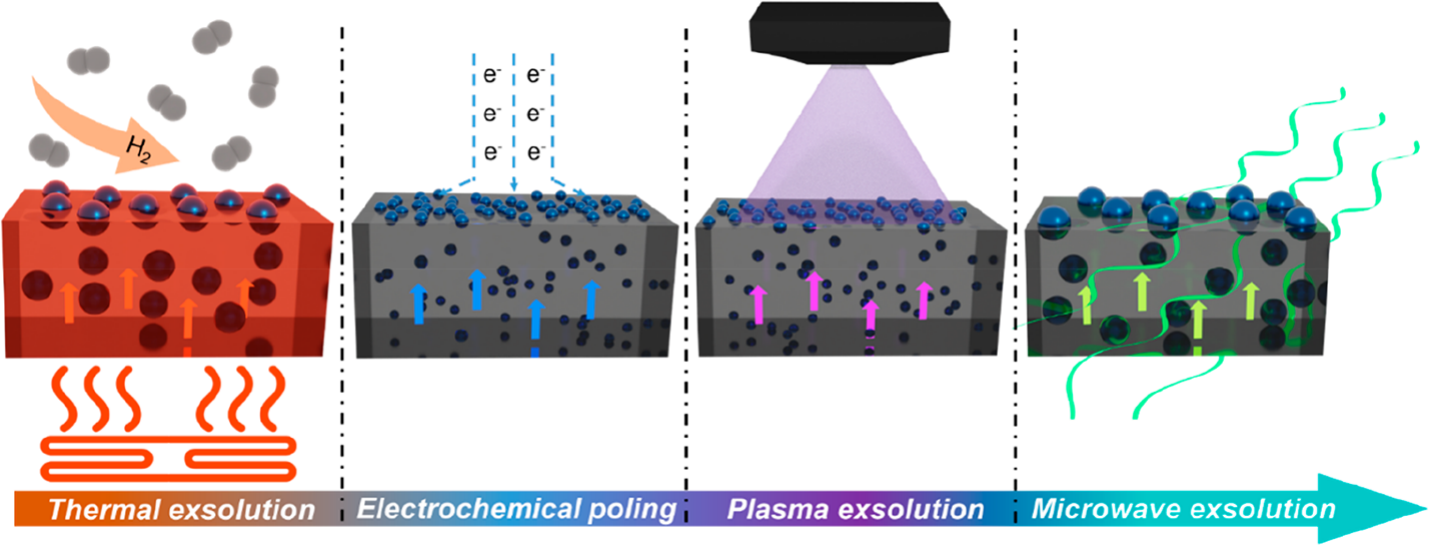
Schematic of the different exsolution methods. The most commonly used thermal exsolution requires medium-high temperatures and a reducing agent flow (typically H_2_) to take place. Recently, two methods have been reported: electrochemical poling and plasma-driven exsolution. Both methods showed high nucleation ratios (and so high NPs populations) and needed short operation times, with no H_2_ needed. Nevertheless, the electrochemical method needs the metal oxide deposited as an electrode, limiting its potential uses. On the other hand, plasma-driven exsolution requires high working vacuums. In this work, we propose an alternative method: MW-driven exsolution. Its low-time consumption, no need for external heating or reducing agent, and the possibility of up-scaling make it a promising exsolution alternative.

Here, we propose microwave radiation (MW) as an alternative for driving the exsolution of metallic nanocatalysts (Figure 1). In previous works, it has been observed that MW radiation can be used to reduce ceramic oxides, as Serra et al. stated in their work, where they accomplished the production of H_2_ via MW-driven water splitting redox cycles.^25^ During oxide reduction, the material undergoes different processes: As the material is MW irradiated, the charges within the material undergo polarization, resulting in an increase in temperature. Upon absorbing a certain amount of energy, which is indicated by an induction temperature (*T*_i_), the interaction between the MW and the material induces a nontypical electronic conductivity, accompanied by a rapid reduction process that releases molecular oxygen from its ionic lattice. This reduced state can remain if the electromagnetic fields are maintained; however, once the MW is discontinued, the material tends to reoxygenate if exposed to oxygen-bearing atmospheres, recovering its original conductivity. Here, we selected La_0.43_Ca_0.37_Ni_0.06_Ti_0.94_O_3−δ_ A-site deficient perovskite (LCTN) to explore the possible MW-mediated exsolution of NPs from complex mixed-oxide hosts. This specific composition can be considered a reference material in this field since it has been employed to demonstrate thermal-, electrochemical-, and plasma-driven exsolution, exhibiting high versatility. This fact brings the possibility of directly comparing the proposed exsolution results with other alternative methods. In addition, the A-site deficiency has been commonly reported as a factor to enhance exsolution, thus triggering an increase in the number of exsolved NPs.^3^ Lastly, lanthanum titanates exhibit high physical and chemical stability.^26^ All of these reasons make LCTN an adequate candidate for these experiments.

This work proves that MW reduction indeed triggers exsolution on LCTN perovskite, with no need for high-temperature heating or H_2_ atmosphere or vacuum. This method also demonstrates low time requirements and even the possibility of controlling specific morphological properties of the exsolved nanoparticles. An important feature of the MW setup used for this exsolution process is the capacity to *in situ* monitor changes in the electrical conductivity while performing the exsolution in powder form, which eases the interpretation of the MW-driven exsolution mechanism. Additionally, MW-driven exsolution is also an easily up-scaling method and can be applied to powder or granulated samples without further oxide manufacturing, bringing the possibility of applying it to a wide variety of alternative metal oxides. Also, with the exsolution results exposed in this work, MW-driven exsolution can be considered to exsolve other metals besides Ni, such as Co, Fe, or Cu, and even for generating alloyed metallic nanoparticles, which have been already obtained via thermal exsolution.^13,14,17,19,21,27,28^ In addition, the exsolved nanoparticles showed catalytic activity for the CO_2_ hydrogenation reaction. The results presented here confirm that MW irradiation provides a promising alternative to thermal exsolution, significantly lowering the working conditions required to functionalize the surface of metal oxides with metallic nanoparticles via conventional exsolution methods.

## Results and Discussion

### Microwave-Driven Exsolution

To test this exsolution method, La_0.43_Ca_0.37_Ni_0.06_Ti_0.94_O_3−δ_ (LCTN, from now on) was selected to evaluate the effects of MW radiation as a reducing agent. This material has been proven to be versatile when subjected to alternative exsolution pathways.^22−24^ In addition, the low conductivity of LCTN makes it an excellent dielectric for MW-driven reduction. This fact and its inherent capability to store energy via polarization modes prevent the appearance of an electric arc between particles. This phenomenon is often seen when a material possesses high electronic conductivity interacting with electromagnetic fields.^25^

For this purpose, a sample of LCTN was placed within the quartz reactor and exposed to focused MW radiation (Figure S1). Simultaneously, the electric conductivity of the sample was *in situ* monitored, enabling a comprehensive understanding of the morphological changes that took place during the oxide reduction and their correlation with the electrochemical properties of the material.

Building upon the previous findings, MW reduction cycles (or pulses) were chosen over continuous wave MW radiation to perform the reductions of the material. Oxygen release predominantly occurs immediately after reaching the *T*_i_.^27^ This means that after applying a given MW power (W g^–1^) for an MW reduction cycle, a few more oxygen vacancies are being formed, so the reduction during the ongoing time would be neglectable. Considering this, several successive pulses should have a higher reductive impact than prolonged exposure times to MW radiation, a fact explored in this work. The reduction treatments were performed under constant N_2_ flow and at room temperature applying between 30 and 50 W g^–1^ of power per reduction cycle. In the applied cycles, the electric field magnitude varies from 59 to 8.56 V/mm in the material, whereas the magnetic field magnitude remains almost constant at 0.02 A/mm due to the field distribution inside the applicator and the position of the sample in a minimum of magnetic field (see details in Figure S2). The magnitude of the electromagnetic fields can be modified by changing the power delivered to the sample.

**Figure 2:**
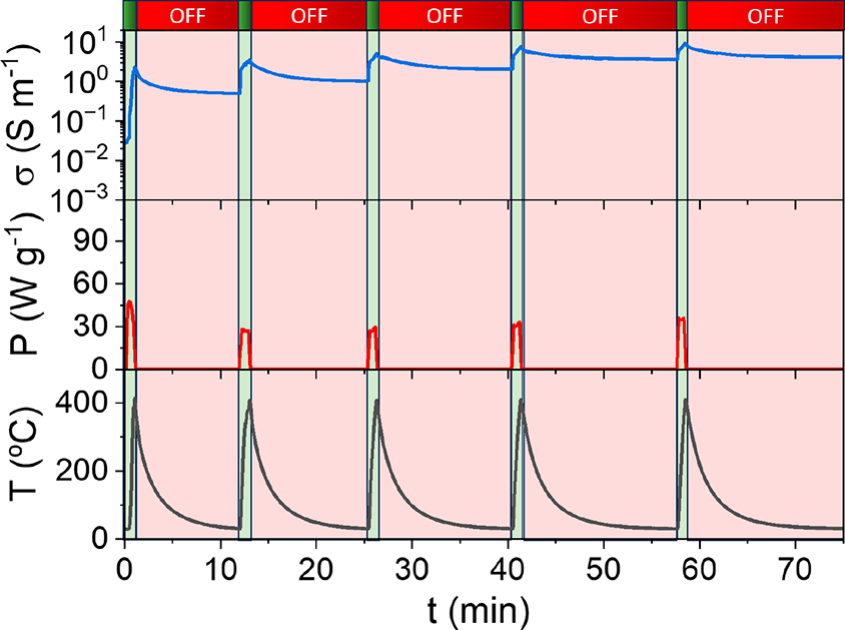
Variations of the LCTN temperature and conductivity when applying 5 consecutive MW reduction cycles, using powers between 30 and 50 W g^–1^.

**Figure 3:**
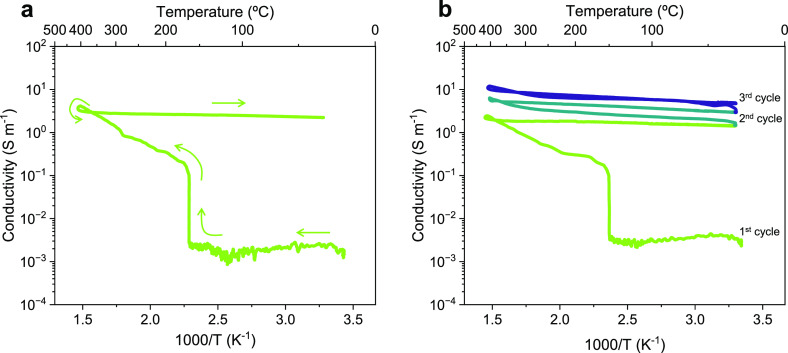
Changes in the electric conductivity of La_0.43_Ca_0.37_Ni_0.06_Ti_0.94_O_3−δ_ after applying (a) 1 and (b) 3 MW reduction pulses. After each cycle, conductivity increases permanently, even after the MW radiation source.

Figure 2 shows an example of a 5-cycle MW treatment performed to drive the exsolution of Ni nanoparticles from LCTN, depicting the MW power used for each MW pulse, the temperature reached during it, and the changes in electric conductivity experienced in the material. As depicted in Figure 2, upon MW radiation, the temperature of the material increases, and once the *T*_i_ is attained (150 °C, approximately), the material exhibits a fast increase in its electric conductivity. Subsequently, when the MW radiation ceases (turned off), the temperature of the material decreases gradually, returning to room temperature in approximately 12 min. This process and the changes suffered by LCTN during the MW heating are also monitored with an inside camera. The recorded videos (Figure S3) showed homogeneous heating of the material, especially in the middle regions of the sample. During the process, the material acquired an unusual shine when the conductivity increased, probably by the interaction of electrons and polarons formed in the reduction process with the electromagnetic field. Interestingly, despite what was observed in previous works,^25^ the material conductivity does not decrease back to the original values when the MW radiation is turned off, indicating that the material is evolving due to the MW radiation influence. To confirm this, the conductivity changes are studied in a typical Arrhenius plot (Figure 3). When the material reaches *T*_i_, it presents a visible increase in its conductivity. It has been reported that this behavior can be ascribed to the interaction of polarons and free electrons located in the ionic lattice with the electromagnetic field.^25^ After a first reduction pulse, once the MW radiation is turned off, the material exhibits high conductivity values, which remain steady upon cooling (Figure 3a). Additionally, as observed in Figure 3b and Figure S4, when several MW cycles are applied to LCTN, its conductivity continues to rise at the end of each cycle. This behavior suggests the appearance of Ni metallic nanoparticles (NPs) on the surface of the material, visibly increasing its conductivity due to the electronic contribution of this metal. The following characterization results will confirm this initial hypothesis, showing the effective exsolution caused by the MW reduction. Nevertheless, it is essential to highlight that the most significant enhancement in conductivity occurs following the initial MW pulse. Consequently, the most crucial alterations in LCTN conductivity occur during the initial cycle, particularly during the liberation of molecular oxygen and the subsequent creation of oxygen vacancies within the perovskite lattice. This exsolution process has the potential to be regulated by precise control over the power supply to the sample, thereby influencing the population and size of surface NPs and, subsequently, altering its catalytic activity.

**Figure 4:**
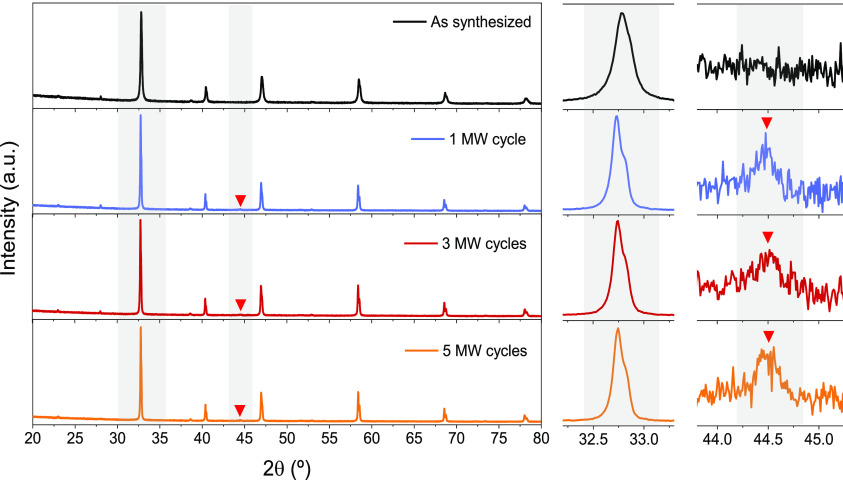
XRD patterns for La_0.43_Ca_0.37_Ni_0.06_Ti_0.94_O_3−δ_ before and after MW reduction treatments, including different cycle number procedures. Ni metallic phase can be appreciated after MW reductions (red triangles).

X-ray diffraction was used to check crystallographic changes induced in the material by the MW treatments. Figure 4 depicts the X-ray diffractograms of the material as synthesized and after 1, 3, and 5 MW cycles. The main peak of the LCTN perovskite phase (2θ ≈ 32.7°) shifts to lower 2θ values after every treatment, which indicates lattice expansion. These results suggest an effective reduction of the material when MW treatments are applied. Similar to the case of conductivity, the larger expansion occurs after the first cycle, the lattice expansion being much less marked with successive cycles; thus, the main reduction happens during the first cycle. In addition, a metallic Ni signal corresponding to the (111) reflection became apparent after MW treatments (2θ = 44.5°). This not only confirms the reduction of the material under MW influence but also sets the stage for possible exsolution of Ni nanoparticles (NPs), together with the permanent increase in the conductivity suffered by the material after every reducing cycle. No further changes in the material can be appreciated, proving the stability of the LCTN in this MW environment.

**Figure 5:**
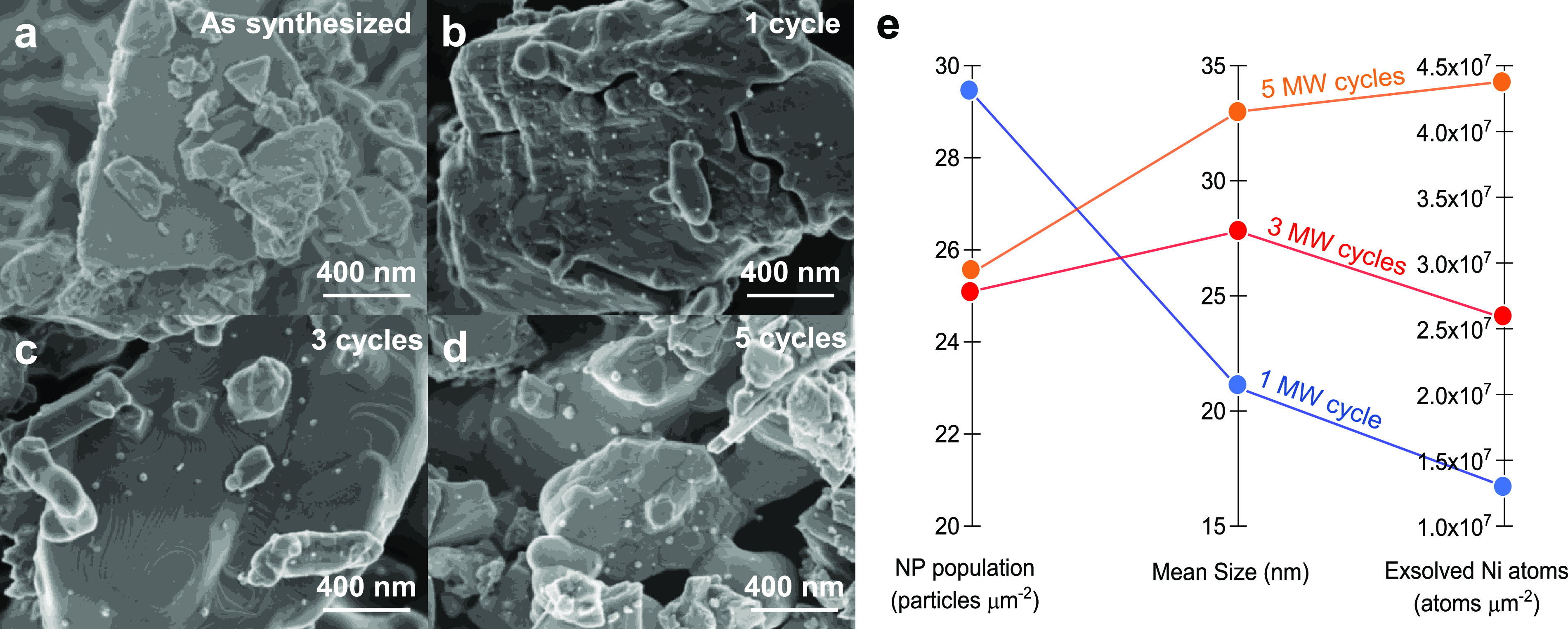
HRFESEM micrographs of La_0.43_Ca_0.37_Ni_0.06_Ti_0.94_O_3−δ_ (a) as-synthesized and after (b) 1, (c), 3 and (d) 5 reduction MW cycles. Nanoparticles emerge over the surface of the material after the application of MW radiation. (e) Comparison between different MW reduction cycles applied to LCTN. Although NP populations do not significantly change after successive cycles, Ni continues exsolving. This fact can be seen in the growing mean sizes of the NPs and the increase in exsolved Ni atoms after different MW exsolution cycles.

Figure 5 shows the SEM micrographs of the material as-synthesized and after different MW reduction cycles to assess the possible morphological changes occurring upon MW exsolution treatment. Exsolution is confirmed as spherical NPs appear on the perovskite surface after 1, 3, and 5 cycles of MW but only after applying the treatments. This fact proves the effective exsolution using MW radiation as a reducing agent. Although the NPs are well dispersed throughout the sample, differences in their dispersion are significant when compared to other alternative exsolution methods (electrochemical, plasma).^22−24^ Our results show that nucleation is less favored with MW reduction, and NPs tend to experience larger growth.

The exsolved nanoparticle size and dispersion were studied after every microwave cycle. Figure S5 depicts the resulting histograms, showing a wider distribution after each microwave treatment. Nevertheless, dispersion does not significantly change with successive reduction cycles: 29.4 particles μm^–2^ with 1 MW reduction cycle, 25.1 particles μm^–2^ after 3 MW cycles, and 25.5 particles μm^–2^ after 5 cycles, but the mean size of the NPs does, as can be seen in Figure 5e (statistical analyses for both NP size and dispersion, can be seen in Figure S6). In fact, NPs size grew after each MW cycle, namely, 21.0, 27.8, and 33.0 nm after 1, 3, and 5 MW cycles, respectively. This progressive growth translates into more Ni atoms exsolution. Thus, although the main exsolution event happens during the first MW pulse, applying more cycles entails ongoing exsolution and thus a modification of the NP size. This fact suggests that specific control over the NP size can be achieved with variation in the MW reduction cycles. However, at a certain point, further MW pulses will not significantly affect the exsolution (see Figure S4) since the supply of Ni atoms is limited by the perovskite lattice stoichiometry. Consequently, particle growth will stop since newer nucleation sites are not being formed after the first MW cycle. It is worth mentioning that although the exsolved nanoparticle dispersion is not as high as for other methods,^22−24^ the low time consumption of reduction cycles (the most of the time is needed for cooling to room temperature) and the possibility of working in the absence of H_2_ (or vacuum) and without external heating make the MW reduction process a promising alternative to thermal exsolution. Nevertheless, future studies need to optimize some parameters to improve the amount of exsolved nanoparticles. For instance, lowering the applied MW power could lead to lower growth and higher nucleation ratios, resulting in larger dispersions.

**Figure 6:**
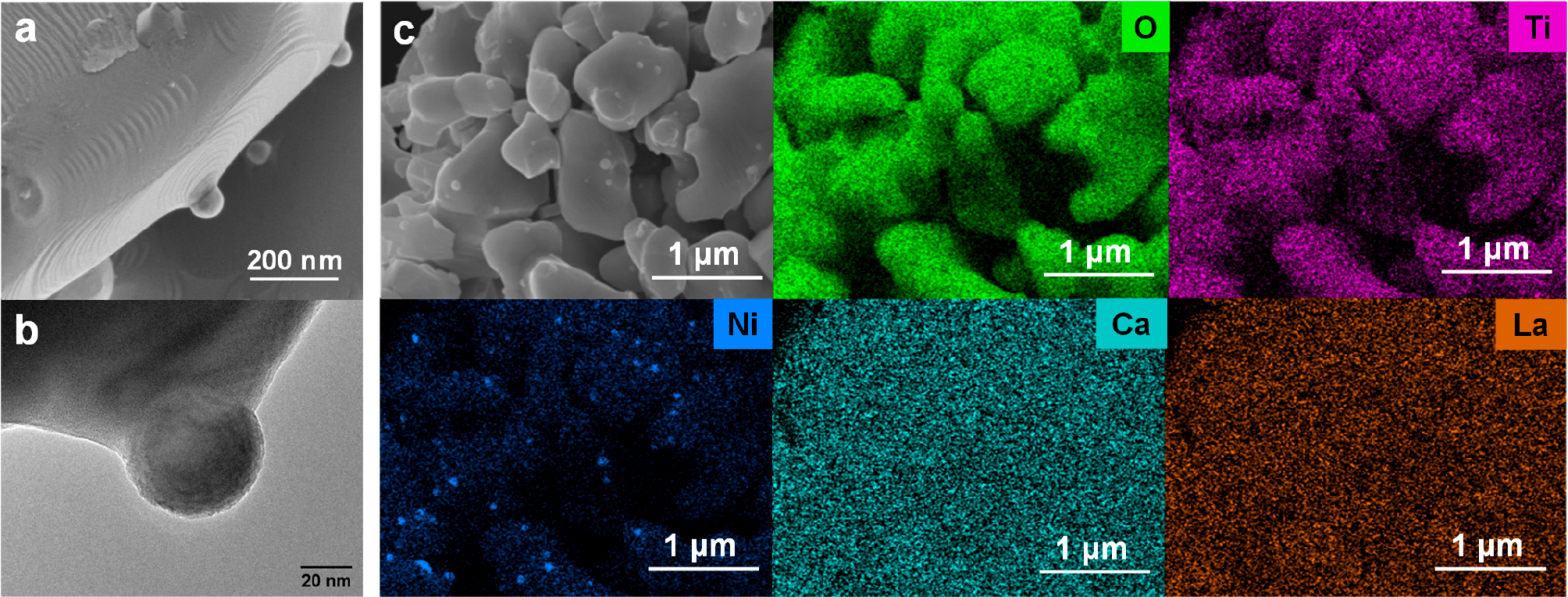
Study of the anchoring of the exsolved NPs after 5 MW reduction cycles applied to La_0.43_Ca_0.37_Ni_0.06_Ti_0.94_O_3−δ_. The micrographs were obtained via (a) HRFESEM and (b) TEM. Lastly, (c) low-magnification HRFESEM micrograph and EDS map analyses were performed after 5 cycle MW exsolution. These results confirm that the exsolved NPs are composed of Ni. Adequate distribution of every atom can be seen along the sample.

Once MW-driven exsolution of NPs is confirmed, TEM studies were carried out to confirm the anchoring of the exsolved nanoparticles (socketing). Figure 6 depicts some isolated exsolved nanoparticles after 5 MW cycles of exsolution (which originated the largest NPs), seen by HRFESEM and TEM. Both Figure 6a and Figure 6b corroborate the anchored nature of these NPs, which clearly emerges from the perovskite lattice. This is a key aspect in exsolution since anchoring provides the nanoparticles with high resistance against sintering or coking. When measuring the interplanar distances with the digital diffraction pattern (DDP) image of these exsolved NPs (Figure S7a), metallic Ni exsolution is confirmed, as the 0.210 nm *d*-spacing corresponds to the (111) plane of the Ni^0^. This fact is consistent with the metallic Ni signals observed via XRD. Also, as can be seen in Figure S7b, the interplanar distances seen in the lattice of the parental oxide (0.270 nm) are undoubtedly close to the main plane of similar perovskite oxides (i.e., 0.270 nm for CaTiO_3_).

Finally, to confirm the composition of the exsolved NPs, EDS studies were performed from HRFESEM analyses. Low-magnification SEM micrograph after 5 MW cycles can be seen in Figure 6c, together with compositional mappings of the sample. Some exsolved NPs can be appreciated in this SEM micrograph. Mapping analyses show a good distribution of the O, Ti, Ni, Ca, and La along the material lattice. Nevertheless, Ni appears clearly as the constituent of the exsolved nanoparticles, which is consistent with the previously analyzed results.

Additionally, XPS analysis (Figure S8) was utilized to investigate the surface composition and nature of the exsolved nanoparticles. However, a challenge arises as the primary La 3d and Ni 2p lines overlap, hindering direct confirmation of the Ni oxidation state. Following MW treatment, all of the signals exhibit a shift in their binding energy. This shift has also been linked to the enrichment of A-site cations, leading to an increased A/B ratio (as evidenced by XPS data), gradual filling of A-site vacancies, and potentially contributing to stabilizing the exsolved particle exsolution.^24^

Moreover, within the orbital 1s region of O, two distinct signals are observed at 351.5 and 529.6 eV, corresponding to water adsorbed on the surface and oxygen present in the ionic lattice, respectively.^24^ In the La region, two signals are detected at 834.7 and 851.5 eV, attributed to the 3d_5/2_ and 3d_3/2_ orbitals of La^3+^, accompanied by additional satellites at 838.5 and 855.30 eV.^30^ The Ca region exhibits two signals at 346.8 and 350.4 eV, corresponding to the 2p_3/2_ and 2p_1/2_ orbitals of Ca^2+^.^31^ Lastly, in the Ti region, two signals representing Ti^4+^ emerge at 458.3 and 464.0 eV, corresponding to the 2p_3/2_ and 2p_1/2_ orbitals, respectively.^32,33^

**Figure 7:**
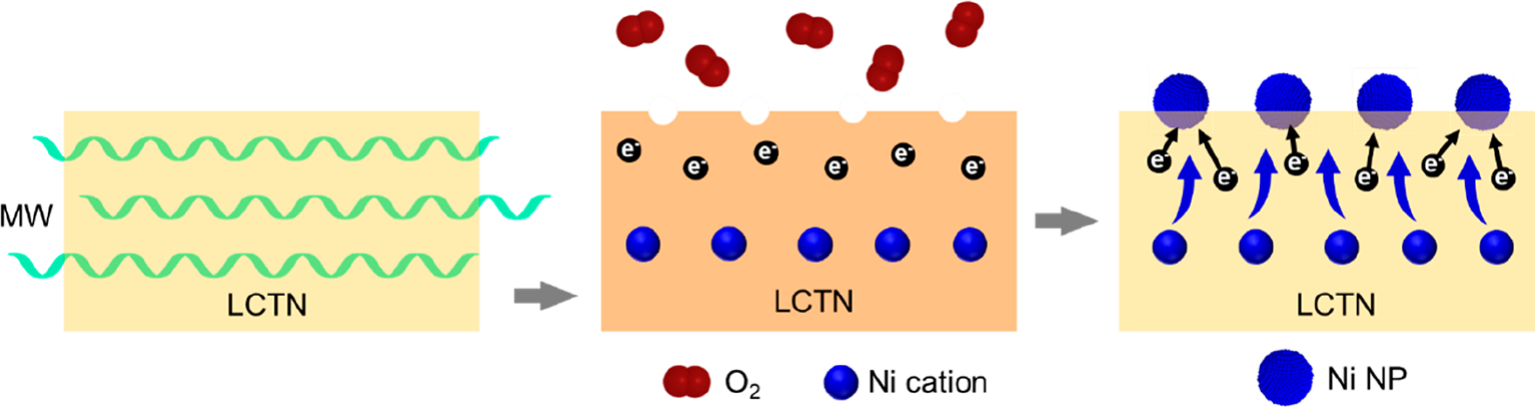
Schematic representation of the mechanism of microwave-driven nanoparticle exsolution. First, LCTN interacts with MW radiation, and the acquired energy is dispelled, leading to an increase in the temperature of the material. When *T*_i_ is reached, an increase in the electronic conductivity can be appreciated in a second step. At this point, MW-induced reduction occurs, leading to the formation of oxygen vacancies and electronic charge carriers. In the final step, these oxygen vacancies are key nucleation sites for metallic NPs that are formed after the migration of Ni cations from the lattice of LCTN to its surface.

Based on all of the obtained results, the mechanism for the microwave-driven exsolution process is ascribed to an electronic-level interaction of the microwaves with the perovskite oxide (Figure 7). First, when the material is exposed to the electromagnetic field, it absorbs microwave radiation and experiences an increase in temperature, resulting from the storage and release of acquired energy. Upon reaching the critical temperature (*T*_i_), the material undergoes microwave-induced reduction, leading to the formation of an oxygen vacancy and two localized electronic charge carriers (known as polarons) for each reduced nickel atom (Ni^2+^ → Ni^0^). At this point, the homologous growth of the electronic-carrier population results in a substantial increase in electronic conductivity (as already seen in Figure 2), owing to the interaction of these charge carriers with the electromagnetic field. Figure S3 illustrates this interaction, explaining the anomalous brightness observed during the acquisition of electronic conductivity to this phenomenon. Consequently, nickel migrates to the material’s surface, capturing the charge carriers generated during the formation of oxygen vacancies and thus transitioning into the metallic state. Upon removing the electromagnetic field, the material gradually returns to room temperature, exhibiting a significantly enhanced overall conductivity compared to its initial state, possibly due to the Ni metallic nanoparticles formed on its surface.^25^

With all this in mind, as oxygen vacancies (V_O_^••^) are preferential nucleation sites for nanoparticle exsolution, formation of these vacancies is key to increasing nucleation rates.^34^ So then, the growth in the number of V_O_^••^ shall increase exsolved NP populations.^34^ Knowing this, it is worth mentioning that *p*O_2_ during the exsolution process is a relevant factor to be taken into account. This *p*O_2_ will determine the observed variation in NP dispersion between MW-driven exsolution and other alternative methods, like electrochemical poling. The notable nucleation differences can be, thereby, ascribed mainly to the lower *p*O_2_ estimated in Myung et al. work (10^–35^ bar) during electrochemical exsolution.^22^ When compared to the *p*O_*2*_ during MW exsolution (estimated in 10^–5^ bar), the V_O_^••^ formation shall be increased with lower *p*O_2_ and thus nucleation sites. This fact may explain the population differences observed between both methods.

### CO_2_ Hydrogenation Reaction Tests

Once MW-driven exsolution was confirmed, the catalytic activity of the exsolved Ni NPs was evaluated. For this purpose, CO_2_ hydrogenation tests were carried out; namely, the reverse water gas shift (RWGS) reaction was selected for characterizing the catalytic nature of the Ni NPs. This moderately endothermic chemical reaction faces competition with the highly exothermic CO_2_ methanation reaction, particularly at low temperatures. Consequently, enhancing the performance and selectivity of the RWGS reaction at lower temperatures is a key challenge for catalyst design.^35^

In this context, Ni has been traditionally associated with methanation. Nevertheless, it has been suggested that its behavior differs depending on whether it is present as an impregnated species or as a metallic state incorporated within the material. Several studies have revealed an interesting distinction in this matter, particularly concerning methanation reactions. In this regard, the oxygen vacancies that arise during exsolution seem to play a critical role, significantly favoring the RWGS reaction over methanation.^36^ In addition, the exsolved metallic Ni over the LCTN surface serves as a catalyst for the desired shift in selectivity toward carbon monoxide production.

**Figure 8:**
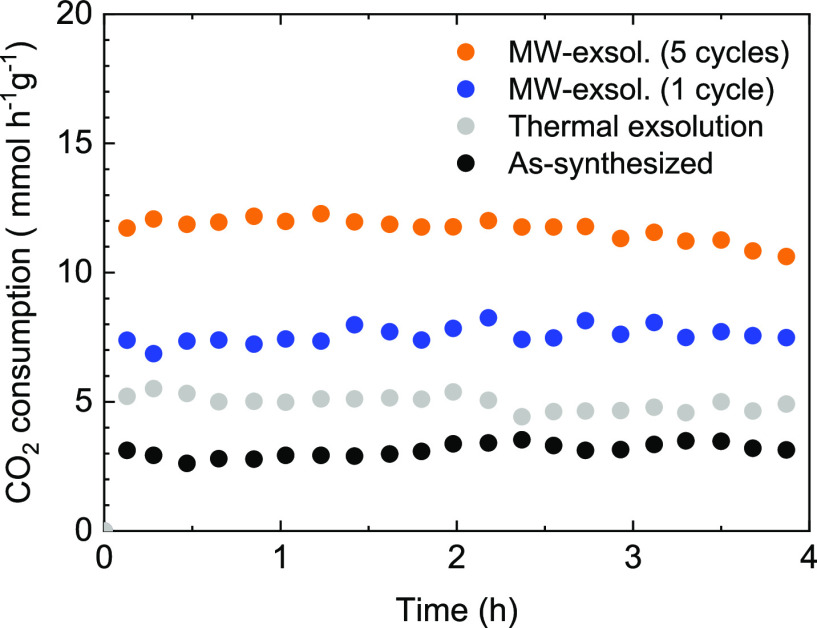
CO_2_ consumption for La_0.43_Ca_0.37_Ni_0.06_Ti_0.94_O_3−δ_ before and after MW reduction treatments, namely, 1 and 5 exsolution cycles. Those two treatments were also compared to an *in situ* thermal exsolution (400 °C, 1 h under 5% H_2_/Ar flow) and with nonexsolved LCTN. All tests were carried out at 400 °C and GHSV = 13971 h^–1^.

In Figure 8, the CO_2_ consumption is evaluated using LCTN as a catalyst but functionalized in two different ways. Specifically, LCTN after thermal exsolution (400 °C, 1 h under 5% H_2_/Ar flow) and LCTN after MW-driven exsolution. For these catalytic tests, LTCN functionalized via 1 and 5 MW-reducing cycles were chosen to observe better the catalytic performance differences between distinct MW exsolution treatments. In addition, nonexsolved LCTN was used as a benchmark. Interestingly, it is observed that the material itself exhibits some catalytic activity, even without exsolution. This activity can be attributed to the presence of Ni in the bulk of the material. After 1 h of thermal exsolution, some scarce NPs (almost negligible number) can be seen over the surface of LCTN (Figure S9), which would explain the slight improvement in CO_2_ consumption observed. In contrast, a notable change in this consumption can be observed with MW-driven exsolution, even after just 1 MW cycle, reaching values around 8 mmol h^–1^ g^–1^ for the 4 h test. This improvement in the catalytic activity becomes even more pronounced after 5 cycles (around 12 mmol h^–1^ g^–1^) and can be related to the higher exsolution rates of Ni atoms compared to 1 MW cycle reduction, seeing that NP populations are very close between MW treatments. The CO_2_ conversions (Figure S10a) are directly proportional to these consumption patterns, with the highest conversion values obtained after multiple exsolution cycles, namely, after 1 MW cycle (9% CO_2_ conversion) and, especially, after 5 MW exsolution pulses (∼14%), that outperforms every other tested material (2% and 4% for as-synthesized and thermally exsolved LCTN, respectively). The higher catalytic activity of MW exsolved materials compared to thermally exsolved LCTN shall be ascribed to the larger amounts of Ni functionalized as metallic Ni in the NPs. Namely, seen is the scarce amount of exsolved NPs after thermal exsolution for 4 h, which are, in addition, at the early stages of their formation, and thereby, the exposed surface of these NPs is lower than the MW exsolved ones. Additionally, the selectivity of the reaction toward CO is consistently above 90% in all cases (Figure S10b).

It is worth mentioning that the thermal exsolution tests were also performed pursuing another goal: stating the differences with the MW exsolution process, which also reached around 400 °C at the end of every reducing cycle. The NP mean sizes obtained with thermal exsolution (around 10 nm, even after 24 h of treatment, Figure S9) are substantially smaller than the ones reached with MW exsolution. This fact states that the observed growth with MW reduction cycles cannot be associated with thermal growth but with an MW radiation effect.

Finally, long-term CO_2_ consumption was studied to evaluate the stability of the 5-cycle exsolved LCTN. Figure S11 shows that CO_2_ consumption remains constant (consistently above 10 mmol h^–1^ g^–1^) even after 60 h of operation except for an initial drop in consumption during the first 10 h. Figure S12a shows the SEM micrographs for the 5-cycle MW-exsolved LCTN after 4 h under the stream, indicating the presence of exsolved NPs on the material’s surface. Exsolved NPs also remain after 60 h tests (Figure S12b), but in addition, some smaller NPs emerge during the reaction due to the presence of H_2_ flow as a reactant. Nevertheless, the larger-sized NPs observed in both micrographs suggest the stability of the MW exsolved Ni NPs during these reaction tests. Also, these experiments revealed the better catalytic performance achieved with MW-driven exsolution compared to a thermal one.

## Conclusions

In this work, we demonstrate the MW-driven exsolution of Ni nanoparticles in the absence of a H_2_ atmosphere or vacuum. This technique enabled us to exsolve Ni NPs from a perovskite host with very short exposure times and no external heating. For these studies, La_0.43_Ca_0.37_Ni_0.06_Ti_0.94_O_3−δ_ A-site deficient perovskite was employed due to its exsolving versatility with alternative exsolution methods (thermal, electrochemical poling, or plasma-driven processes). The reduction was carried out with successive MW pulses (1, 3, and 5 MW cycles), and it translated into an effective exsolution of Ni metallic nanoparticles. The presence of well-dispersed nanoparticles was observed even after a single cycle of MW irradiation, highlighting the short duration required for this exsolution method to take effect. Although the main reduction happens with the first cycle, NPs growth occurred after applying several successive reduction pulses. This fact showed an ongoing exsolution of metallic Ni atoms after several cycles. Nevertheless, populations do not significantly change, suggesting that growth rates are favored over nucleation processes. The exsolved materials were tested as catalysts for RWGS reaction, exhibiting increased catalytic activity after MW-driven exsolution, even outperforming the thermally exsolved LCTN and showing notable stability of the NPs after 60 h of reaction. In summary, this work presents an exsolution method, with low energy and time requirements, with the possibility of easy up-scaling employing powder materials. MW-driven exsolution will facilitate overcoming some disadvantages of classic thermal exsolution, expanding the realm of exsolution methods with high versatility in energy applications for green fuel production.

## Experimental Methods

### Materials Synthesis

Powders of La_0.43_Ca_0.37_Ni_0.06_Ti_0.94_O_3−δ_ were synthesized via the solid-state reaction (SSR) method. Appropriate amounts of the metal precursors were mixed and milled for 24 h in acetone, namely, La_2_O_3_ (99.99%, Aldrich), CaCO_3_ (99%, Aldrich), TiO_2_ (99.8%, Aldrich), and Ni(NO_3_)·6H_2_O (98%, Alfa-Aesar). Before weighing, both La_2_O_3_ and TiO_2_ were dried for 1 h at 250 °C. After this initial milling, mixed powders were ground in an agate mortar and sieved below 200 μm. These fine powders were subjected to an initial sintering, 1000 °C for 12 h. The resulting powders were ground again and pressed into a pellet under 30 kN for 3 min. Then, the pellet was sintered for 24 h at 1400 °C. Finally, the obtained solid was milled for 72 h in acetone and sieved below 200 μm.

### Microwave-Driven Reduction

The reduction of ceramic materials was carried out within a tubular volume with a diameter of 9.8 mm and a height of 15 mm inside a quartz reactor equipped with a quartz frit in a 150 mL min^–1^ dry N_2_ flow. This was achieved by inserting the quartz reactor in an MW cylindrical cavity operating in the TE111 transverse electric mode, specifically around the ISM (industrial, scientific, and medical) frequency of 2 GHz. The MW cavity was designed with open apertures in the top, bottom, and side walls to facilitate the introduction of the tubular quartz reactor containing the sample, as well as the positioning of antennas for MW power coupling and process monitoring.

The MW irradiation setup made use of a 120 W solid-state MW amplifier (RCA2026U50, RFcoreLtd. from 2.2 to 2.6 GHz), which was driven by the oscillator and receiver of a network analyzer (Rohde & Schwarz ZVRE), along with a sophisticated MW control system to ensure a precise level of MW radiation delivered to the material. The MW control system employed a variable coupling device (coextensive request with variable penetration) and a dual directional coupler (Meca Electronics, model 722-40 1950 W) to measure and adjust the reflected signals of the MW cavity as a function of frequency and temperature. To determine the volumetric temperature of the material, the surface temperature of the quartz reactor was measured using an IR pyrometer (Optris CT-Laser LT) according to the procedure described in ref (27).

A mass spectrometer (Omnistar Balzers) was connected to the side of the tubular quartz reactor to measure the exhaust gas composition during the experiments. To quantify the released gas species from the material, the gases in the mass spectrometer were calibrated (O_2,_ H_2_, CH_4_, CO, and CO_2_). Calibration gas bottles (purity 5.0) were purchased from Linde.

Additionally, the MW cavity perturbation technique (MCPT) was used to measure *in situ* the material’s dielectric properties, providing valuable insights into the reduction process. The method involved introducing a weak frequency sweep signal into a resonant cavity for MW radiation and carefully monitoring alterations in the resonance characteristics and MW losses. These variations yielded the parameters required for both the real and imaginary components of permittivity determination, which are directly associated with AC conductivity. By analyzing these parameters, a comprehensive understanding of the reduction process and the material’s conductivity could be attained, contributing to a more profound knowledge of its behavior under MW irradiation. Crucially, the MCPT setup was designed to minimize any interference with the MW electromagnetic fields encompassing the specimen, thereby facilitating precise measurements of the conductivity and permittivity.

The materials were reduced through a series of measurements involving varying numbers of reduction cycles. Initially, the materials were subjected to MW radiation to heat them until they reached a (certain) *T*_i_. Once the desired temperature was attained, the power supplied was adjusted to approximately 40 W g^–1^. Subsequently, as the material reached a temperature of 400 °C, the MW radiation was turned off, followed by a gradual decrease in temperature. The next reduction cycle was initiated after the material had cooled down to room temperature.

### Physicochemical Characterization

X-ray diffractometry (XRD) was employed to study the crystal phases before and after the reduction treatments. For that purpose, a fast diffractometer from PANalytical CubiX was used, with Cu Kα_1,2_ radiation source. For this work, morphological characterization was key, and to unveil changes suffered by the perovskite after applying MW, electron microscopies were performed, concretely, high-resolution field emission scanning electron microscopy (HRFESEM) and transmission electron microscopy (TEM). SEM studies were carried out using a Zeiss GeminiSEM 500. The obtained micrographs were useful for acquiring dispersion and size of the obtained nanoparticles, both analyzed with ImageJ software^29^ (1.52a). To calculate the exsolved Ni atoms, calculations were done as follows:
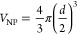
1
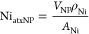
2where *V*_NP_ is the volume of the exsolved nanoparticle, based in its calculated mean diameter (*d*); Ni_atxNP_ the exsolved Ni atoms in a nanoparticle; ρ_Ni_ the Ni density (8900 kg m^–3^); and *A*_Ni_ the atomic mass of Ni (58.693 u) in kg. Using the exsolved NPs populations, Ni atoms μm^–2^ can be finally calculated.

On the other hand, compositional and crystallographic parameters of the nanoparticles (or the perovskite backbone) were explored using high-resolution TEM instrument, specifically with a JEM 2100F 200 kV field microscope. To calculate interplanar distances out of TEM micrographs, ImageJ software was also employed in order to obtain FFT and inverse FFT images. Energy dispersive X-ray spectroscopy (EDS) was also helpful, and these analyses were done with an Oxford Instruments EDS X-Max 80.

Finally, X-ray photoelectron spectroscopy (XPS) was also performed to analyze the materials before and after MW exposure. These analyses used a SPECS spectrometer with a monochromatic Al Kα source and an MCD-9 detector.

### CO_2_ Hydrogenation Reaction Tests

The CO_2_ hydrogenation (CO_2_ + H_2_ → CO + H_2_O) activity of the prepared materials was investigated by using a fixed-bed quartz reactor with an inner diameter of 10 mm. The materials were prepared with particle sizes ranging between 200 and 400 μm. A total of 500 mg of fresh material, mixed with 1.1 g of SiC, was vertically loaded onto the quartz frit in the reactor. SiC was incorporated to enhance heat transfer and improve flow rates within the system. This improvement of heat distribution is key in exothermal reactions and avoids the appearance of hot spots. Due to its thermal stability and inertness, no side effects during the catalytic test are expected due to the presence of SiC.

In a typical run, the temperature was raised to the reaction temperature, 400°C, in an inert atmosphere of argon (Ar). Subsequently, the CO_2_ (Corgon 15) and H_2_ reaction gases, along with an internal standard, were introduced into the system in a ratio of 5:20:1:33 (CO_2_:H_2_:N_2_:Ar). The time–space velocity (GHSV) was set at 13 971 h^–1^. Time on stream is typically 4.5 h, and long-term operation was evaluated during 60 h.

To ensure real-time monitoring of the flue gas composition, a gas chromatograph (Bruker 450GC) was employed. The CO_2_ conversion, CO selectivity, and CO yield were calculated by
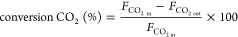
3
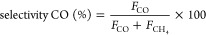
4
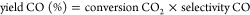
5
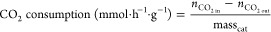
6where *F* is the volumetric flow rate of CO_2_ or CH_4_ (mL/min), *n* is the molar flow rate of CO_2_ (mmol·h^–1^), and the mass_cat_ is the mass of the catalyst loaded into the reactor expressed in grams.

## Supporting Information Available

Setup schematic for microwave reductions, snapshots of the reduction process, nanoparticle size distribution histograms, HRTEM and DDP analyses, XPS spectra, HRFESEM micrographs for thermal exsolution of LCTN, CO_2_ conversion and selectivity comparatives between exsolved materials (including long-term stability), and post-mortem HRFESEM micrographs (PDF)

## References

[ref1] Liu L.; Corma A. Metal Catalysts for Heterogeneous Catalysis: From Single Atoms to Nanoclusters and Nanoparticles. Chem. Rev. 2018, 118 (10), 4981–5079. 10.1021/acs.chemrev.7b00776. 29658707 PMC6061779

[ref2] Zhang J.; Gao M. M.-R.; Luo J.-L. In Situ Exsolved Metal Nanoparticles: A Smart Approach for Optimization of Catalysts. Chem. Mater. 2020, 32 (13), 5424–5441. 10.1021/acs.chemmater.0c00721.

[ref3] Sun X.; Chen H.; Yin Y.; Curnan M. T.; Han J. W.; Chen Y.; Ma Z. Progress of Exsolved Metal Nanoparticles on Oxides as High Performance (Electro)Catalysts for the Conversion of Small Molecules. Small 2021, 17 (10), 2005383 10.1002/smll.202005383. 33538089

[ref4] Kousi K.; Tang C.; Metcalfe I. S.; Neagu D. Emergence and Future of Exsolved Materials. Small 2021, 17 (21), 2006479 10.1002/smll.202006479. 33787009

[ref5] Bhalla A. S.; Guo R.; Roy R. The Perovskite Structure—a Review of Its Role in Ceramic Science and Technology. Materials Research Innovations 2000, 4 (1), 3–26. 10.1007/s100190000062.

[ref6] Sun C.; Alonso J. A.; Bian J. Recent Advances in Perovskite-Type Oxides for Energy Conversion and Storage Applications. Adv. Energy Mater. 2021, 11 (2), 2000459 10.1002/aenm.202000459.

[ref7] Kim J. H.; Kim J. K.; Liu J.; Curcio A.; Jang J.-S.; Kim I.-D.; Ciucci F.; Jung W. Nanoparticle Ex-Solution for Supported Catalysts: Materials Design, Mechanism and Future Perspectives. ACS Nano 2021, 15 (1), 81–110. 10.1021/acsnano.0c07105. 33370099

[ref8] Cao T.; Kwon O.; Gorte R. J.; Vohs J. M. Metal Exsolution to Enhance the Catalytic Activity of Electrodes in Solid Oxide Fuel Cells. Nanomaterials 2020, 10 (12), 2445 10.3390/nano10122445. 33297343 PMC7762234

[ref9] Neagu D.; Irvine J. T. S.; Wang J.; Yildiz B.; Opitz A. K.; Fleig J.; Wang Y.; Liu J.; Shen L.; Ciucci F.; Rosen B. A.; Xiao Y.; Xie K.; Yang G.; Shao Z.; Zhang Y.; Reinke J.; Schmauss T. A.; Barnett S. A.; Maring R.; Kyriakou V.; Mushtaq U.; Tsampas M. N.; Kim Y.; O’Hayre R.; Carrillo A. J.; Ruh T.; Lindenthal L.; Schrenk F.; Rameshan C.; Papaioannou E. I.; Kousi K.; Metcalfe I. S.; Xu X.; Liu G. Roadmap on Exsolution for Energy Applications. Journal of Physics: Energy 2023, 5 (3), 031501 10.1088/2515-7655/acd146.

[ref10] Lee J.; Bae M.; Bae J. Effects of Preparation Method on Exsolution and Alloy Formation in a PtRu Bimetallic Catalyst for Hydrogen Production via Diesel Reforming: Impregnation versus Combustion Synthesis. Int. J. Hydrogen Energy 2022, 47, 29327 10.1016/j.ijhydene.2022.06.261.

[ref11] Carrillo A. J.; Navarrete L.; Laqdiem M.; Balaguer M.; Serra J. M. Boosting Methane Partial Oxidation on Ceria through Exsolution of Robust Ru Nanoparticles. Materials Advances 2021, 2 (9), 2924–2934. 10.1039/D1MA00044F.

[ref12] Meng X.; Wang Y.; Zhao Y.; Zhang T.; Yu N.; Chen X.; Miao M.; Liu T. In-Situ Exsolution of Nanoparticles from Ni Substituted Sr_2_Fe_1.5_Mo_0.5_O_6_ Perovskite Oxides with Different Ni Doping Contents. Electrochim. Acta 2020, 348, 136351 10.1016/j.electacta.2020.136351.

[ref13] Lv H.; Lin L.; Zhang X.; Li R.; Song Y.; Matsumoto H.; Ta N.; Zeng C.; Fu Q.; Wang G.; Bao X. Promoting Exsolution of RuFe Alloy Nanoparticles on Sr_2_Fe_1.4_Ru_0.1_Mo_0.5_O_6−δ_ via Repeated Redox Manipulations for CO_2_ Electrolysis. *Nature*. Communications 2021, 12 (1), 5665 10.1038/s41467-021-26001-8. PMC8476569 34580312

[ref14] López-García A.; Almar L.; Escolástico S.; Hungría A. B.; Carrillo A. J.; Serra J. M. Tuning Ternary Alloyed Nanoparticle Composition and Morphology by Exsolution in Double Perovskite Electrodes for CO_2_ Electrolysis. ACS Applied Energy Materials 2022, 5 (11), 13269–13283. 10.1021/acsaem.2c01829.

[ref15] Kim Y. H.; Kang Y.; Jo S.; Jeong H.; Neagu D.; Myung J. Shape-Shifting Nanoparticles on a Perovskite Oxide for Highly Stable and Active Heterogeneous Catalysis. Chemical Engineering Journal 2022, 441, 136025 10.1016/j.cej.2022.136025.

[ref16] Ansari H.; Bass A. S.; Ahmad N.; Birss V. I. Unraveling the Evolution of Exsolved Fe-Ni Alloy Nanoparticles in Ni-Doped La_0.3_Ca_0.7_Fe_0.7_Cr_0.3_O_3-δ_ and Their Role in Enhancing CO_2_ -CO Electrocatalysis. J. Mater. Chem. A 2022, 10, 2280 10.1039/D1TA07552G.

[ref17] Santaya M.; Troiani H. E.; Caneiro A.; Mogni L. V. Ternary Ni–Co–Fe Exsolved Nanoparticles/Perovskite System for Energy Applications: Nanostructure Characterization and Electrochemical Activity. ACS Applied Energy Materials 2020, 3 (10), 9528–9533. 10.1021/acsaem.0c01997.

[ref18] Neagu D.; Oh T.-S.; Miller D. N.; Ménard H.; Bukhari S. M.; Gamble S. R.; Gorte R. J.; Vohs J. M.; Irvine J. T. S. Nano-Socketed Nickel Particles with Enhanced Coking Resistance Grown in Situ by Redox Exsolution. Nat. Commun. 2015, 6 (1), 8120 10.1038/ncomms9120. 26360910 PMC4579408

[ref19] Lv H.; Lin L.; Zhang X.; Song Y.; Matsumoto H.; Zeng C.; Ta N.; Liu W.; Gao D.; Wang G.; Bao X. In Situ Investigation of Reversible Exsolution/Dissolution of CoFe Alloy Nanoparticles in a Co-Doped Sr_2_Fe_1.5_Mo_0.5_O_6– δ_ Cathode for CO 2 Electrolysis. Adv. Mater. 2020, 32 (6), 1906193 10.1002/adma.201906193. 31894628

[ref20] Santaya M.; Jiménez C.; Troiani H. E.; Carbonio E. A.; Arce M. D.; Toscani L. M.; Garcia-Diez R.; Wilks R. G.; Knop-Gericke A.; Bär M.; Mogni L. V. Tracking the Nanoparticle Exsolution/Reoxidation Processes of Ni-Doped SrTi_0.3_Fe_0.7_O_3-δ_ Electrodes for Intermediate Temperature Symmetric Solid Oxide Fuel Cells. J. Mater. Chem. A 2022, 10, 15554 10.1039/D2TA02959F.

[ref21] Guo J.; Cai R.; Cali E.; Wilson G. E.; Kerherve G.; Haigh S. J.; Skinner S. J. Low-Temperature Exsolution of Ni–Ru Bimetallic Nanoparticles from A-Site Deficient Double Perovskites. Small 2022, 18, 2107020 10.1002/smll.202107020. 35182013

[ref22] Myung J.; Neagu D.; Miller D. N.; Irvine J. T. S. Switching on Electrocatalytic Activity in Solid Oxide Cells. Nature 2016, 537 (7621), 528–531. 10.1038/nature19090. 27548878

[ref23] Kyriakou V.; Sharma R. K.; Neagu D.; Peeters F.; De Luca O.; Rudolf P.; Pandiyan A.; Yu W.; Cha S. W.; Welzel S.; van de Sanden M. C. M.; Tsampas M. N. Plasma Driven Exsolution for Nanoscale Functionalization of Perovskite Oxides. Small Methods 2021, 5, 2100868 10.1002/smtd.202100868. 34928018

[ref24] Khalid H.; Haq A. ul; Alessi B.; Wu J.; Savaniu C. D.; Kousi K.; Metcalfe I. S.; Parker S. C.; Irvine J. T. S.; Maguire P.; Papaioannou E. I.; Mariotti D. Rapid Plasma Exsolution from an A-site Deficient Perovskite Oxide at Room Temperature. Adv. Energy Mater. 2022, 12 (45), 2201131 10.1002/aenm.202201131.

[ref25] Serra J. M.; Borrás-Morell J. F.; García-Baños B.; Balaguer M.; Plaza-González P.; Santos-Blasco J.; Catalán-Martínez D.; Navarrete L.; Catalá-Civera J. M. Hydrogen Production via Microwave-Induced Water Splitting at Low Temperature. Nature Energy 2020, 5 (11), 910–919. 10.1038/s41560-020-00720-6.

[ref26] Kyriakou V.; Neagu D.; Papaioannou E. I.; Metcalfe I. S.; van de Sanden M. C. M.; Tsampas M. N. Co-Electrolysis of H2O and CO2 on Exsolved Ni Nanoparticles for Efficient Syngas Generation at Controllable H_2_/CO Ratios. Applied Catalysis B: Environmental 2019, 258 (July), 117950 10.1016/j.apcatb.2019.117950.

[ref27] Carrillo A. J.; Serra J. M. Exploring the Stability of Fe–Ni Alloy Nanoparticles Exsolved from Double-Layered Perovskites for Dry Reforming of Methane. Catalysts 2021, 11 (6), 741 10.3390/catal11060741.

[ref28] Wang Y.; Liu T.; Li M.; Xia C.; Zhou B.; Chen F. Exsolved Fe-Ni Nano-Particles from Sr_2_Fe_1.3_Ni_0.2_Mo_0.5_O_6_ Perovskite Oxide as a Cathode for Solid Oxide Steam Electrolysis Cells. J. Mater. Chem. A 2016, 4 (37), 14163–14169. 10.1039/C6TA06078A.

[ref29] Schneider C. A.; Rasband W. S.; Eliceiri K. W. NIH Image to ImageJ: 25 Years of Image Analysis. Nat. Methods 2012, 9 (7), 671–675. 10.1038/nmeth.2089. 22930834 PMC5554542

[ref30] Shkerin S. N.; Kuznetsov M. V.; Kalashnikova N. A. X-Ray Photoelectron Spectroscopy of the Surface of Solid Electrolyte La0.88Sr0.12Ga0.82Mg0.18O3-α. Russian Journal of Electrochemistry 2003, 39 (6), 591–599. 10.1023/A:1024140924902.

[ref31] Demri B.; Muster D. XPS Study of Some Calcium Compounds. Journal of Materials Processing Technol. 1995, 55 (3–4), 311–314. 10.1016/0924-0136(95)02023-3.

[ref32] Gonbeau D.; Guimon C.; Pfister-Guillouzo G.; Levasseur A.; Meunier G.; Dormoy R. XPS Study of Thin Films of Titanium Oxysulfides. Surf. Sci. 1991, 254 (1–3), 81–89. 10.1016/0039-6028(91)90640-E.

[ref33] Liu Y. Y.; Qian L. Q.; Guo C.; Jia X.; Wang J. W.; Tang W. H. Natural Superhydrophilic TiO_2_/SiO_2_ Composite Thin Films Deposited by Radio Frequency Magnetron Sputtering. J. Alloys Compd. 2009, 479 (1–2), 532–535. 10.1016/j.jallcom.2008.12.125.

[ref34] Wang J.; Yang J.; Opitz A. K.; Bowman W.; Bliem R.; Dimitrakopoulos G.; Nenning A.; Waluyo I.; Hunt A.; Gallet J.-J.; Yildiz B. Tuning Point Defects by Elastic Strain Modulates Nanoparticle Exsolution on Perovskite Oxides. Chem. Mater. 2021, 33 (13), 5021–5034. 10.1021/acs.chemmater.1c00821.

[ref35] Zhu M.; Ge Q.; Zhu X. Catalytic Reduction of CO_2_ to CO via Reverse Water Gas Shift Reaction: Recent Advances in the Design of Active and Selective Supported Metal Catalysts. Transactions of Tianjin University 2020, 26 (3), 172–187. 10.1007/s12209-020-00246-8.

[ref36] Rodrigues M. T.; Zonetti P. C.; Alves O. C.; Sousa-Aguiar E. F.; Borges L. E. P.; Appel L. G. RWGS Reaction Employing Ni/Mg(Al,Ni)O – The Role of the O Vacancies. Applied Catalysis A: General 2017, 543 (June), 98–103. 10.1016/j.apcata.2017.06.026.

