# Supplementary material for: Microwave-Driven Exsolution of Ni Nanoparticles in A-Site Deficient Perovskites
Source: ACS Nano. 2023 Nov 17;17(23):23955–64. doi: 10.1021/acsnano.3c08534 (PMC10722607; doi:10.1021/acsnano.3c08534)
Supplement: Supplementary file 1 — nn3c08534_si_001.pdf [file nn3c08534_si_001.pdf]

Supporting Information for

Microwave-driven exsolution of Ni nanoparticles in A-site deficient perovskites

Andrés López-García[1,+], Aitor Domínguez-Saldaña[1,+], Alfonso J. Carrillo[1], Laura Navarrete[1], Maria I. Valls[1], Beatriz García-Baños[2], Pedro J. Plaza-Gonzalez[2], José M. Catala-Civera[2*], José M. Serra[1*]

[1]*Instituto de Tecnología Química (Universitat Politècnica de València-Consejo Superior de Investigaciones Científicas), Av. dels Tarongers, 46022 València, Spain.*

[2]*Instituto ITACA (Universitat Politècnica de València), Camí de Vera, 46022 València, Spain*

[*]Corresponding authors: jmcatala@dcom.upv.es, jmserra@itq.upv.es

[+]Equally contributing.

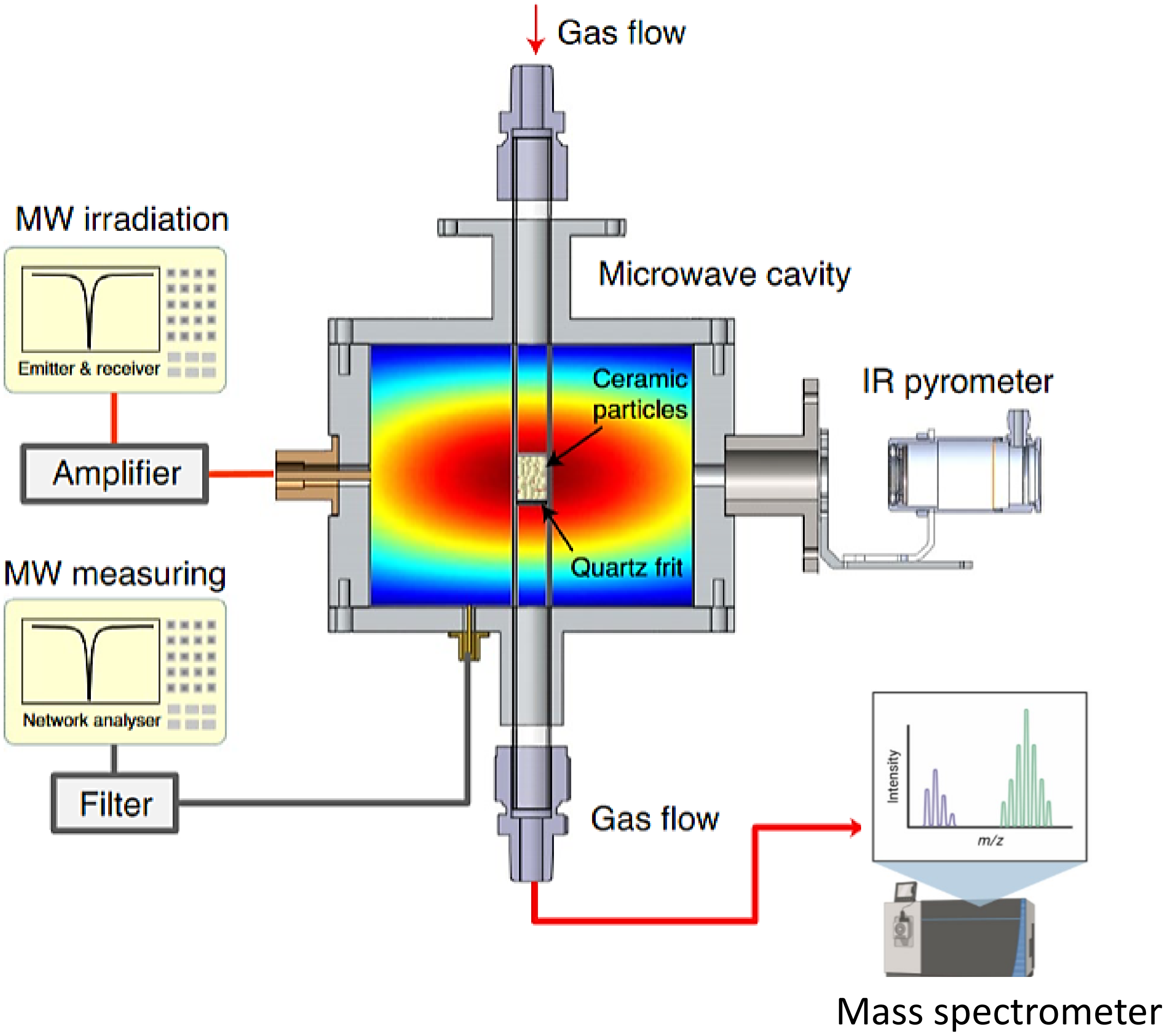

Figure S1. Schematic representation of the set-up used for MW driven reductions of LCTN.

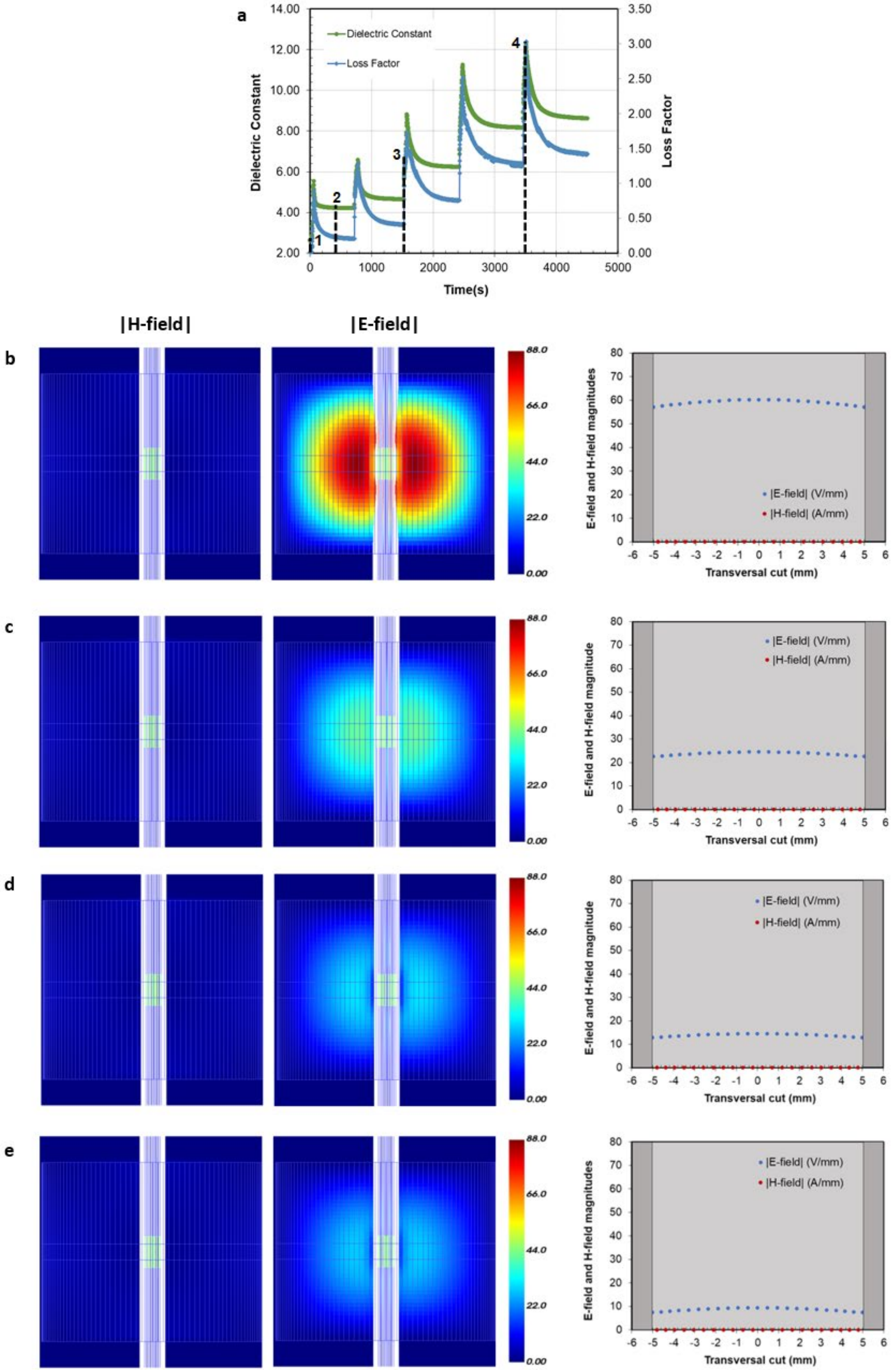

Figure S2. **(a)** Sample's dielectric properties (dielectric constant and loss factor) during reduction cycles. The marked cases represent the four selected stages to represent E-field and H-field variations. **(b)** Field distribution at Stage 1, T = 29 ºC, **(c),** Stage 2, T = 50 ºC, (**d)** Stage 3, T = 206 ºC, and **(e)** Stage 4, T = 410 ºC.

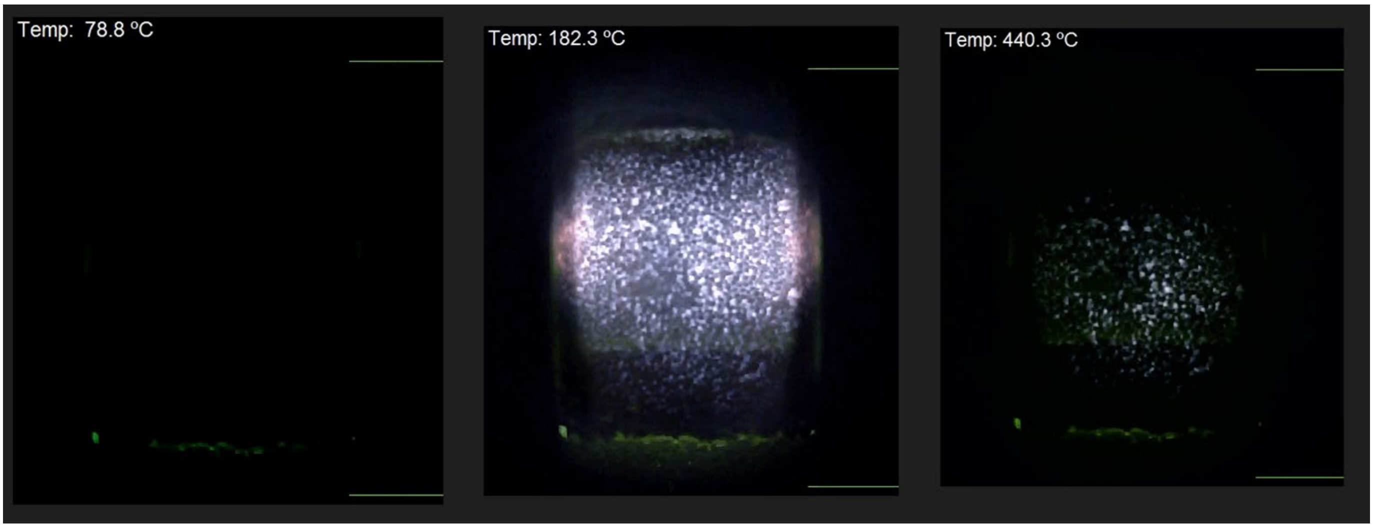

Figure S3. Inside view of the quartz reactor during the MW reduction pulse. Images taken from a recorded video show the material before the T_i (78.8 ºC), when T_i is reached (182.3 ºC) and after turning MW off when the cooldown to room temperature begins (440.3 ºC).

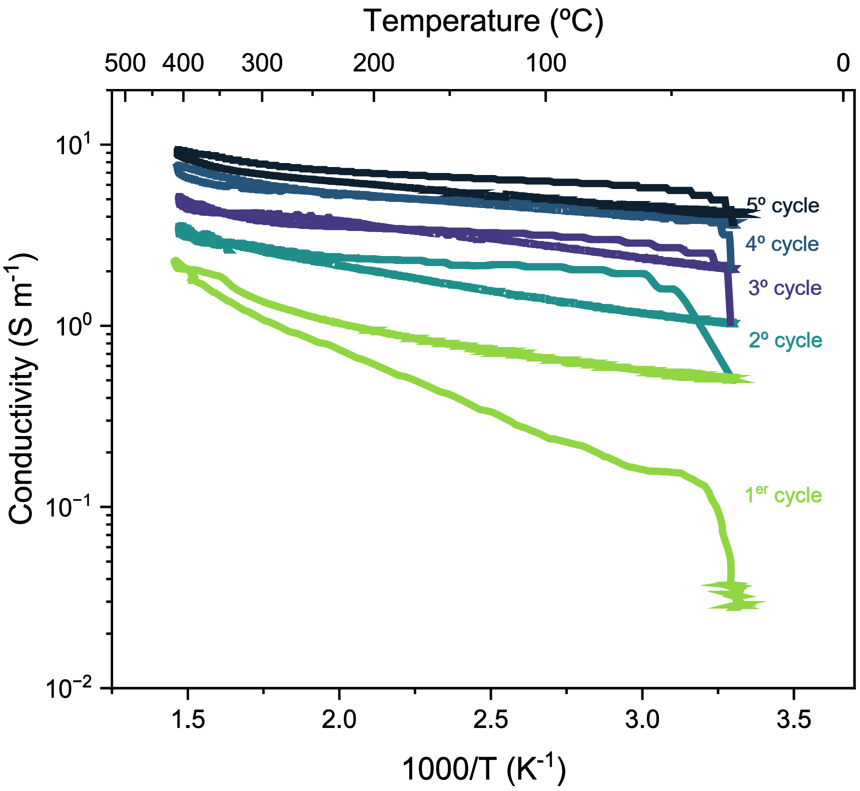

Figure S4. Changes in the electric conductivity of $La_{0.43}Ca_{0.37}Ni_{0.06}Ti_{0.94}O_{3-\delta}$ after applying 5 MW reduction pulses. After each cycle, conductivity increases permanently, even after turning down the MW radiation source.

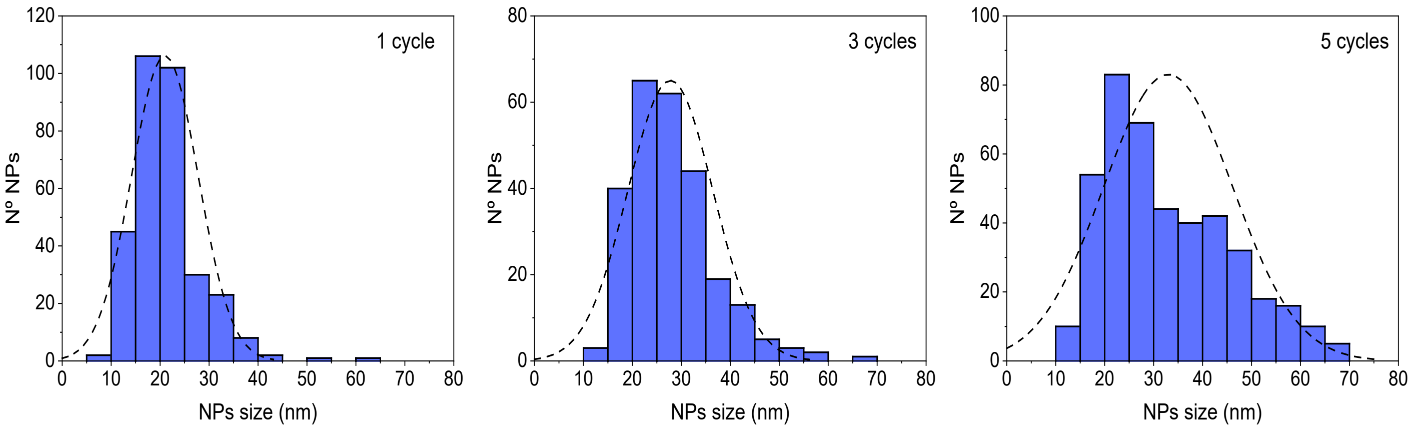

Figure S5. Histograms for the size distribution of the exsolved nanoparticles from $La_{0.43}Ca_{0.37}Ni_{0.06}Ti_{0.94}O_{3-\delta}$ after 1, 3 and 5 MW reduction cycles.

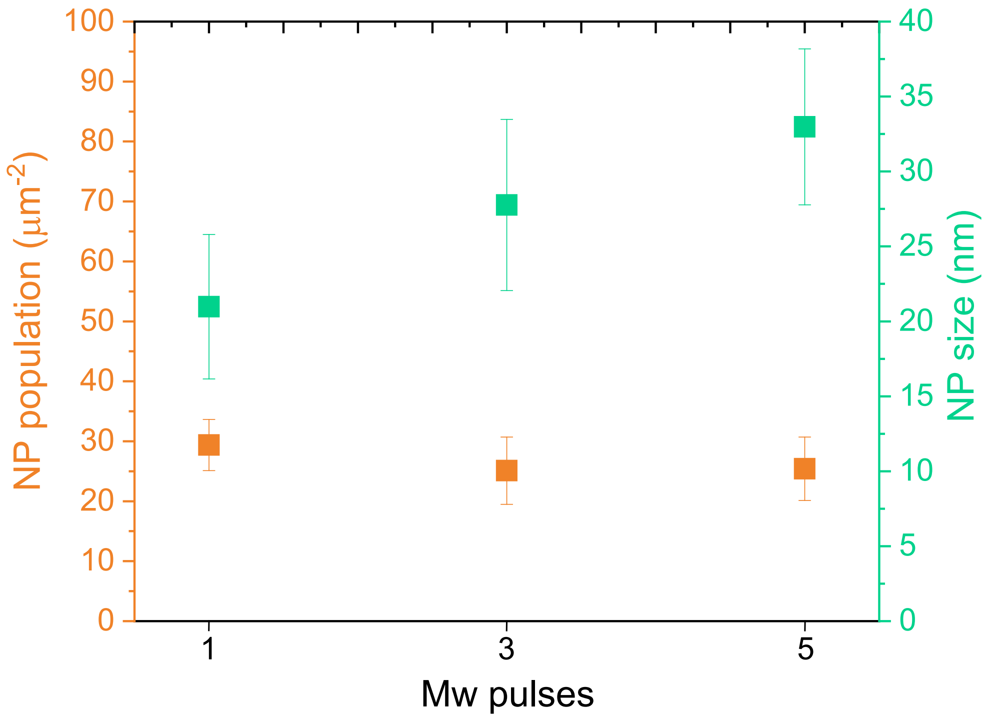

Figure S6. Statistical analyses for NP size and dispersion after MW-driven exsolution with different number of MW pulses (1, 3 and 5).

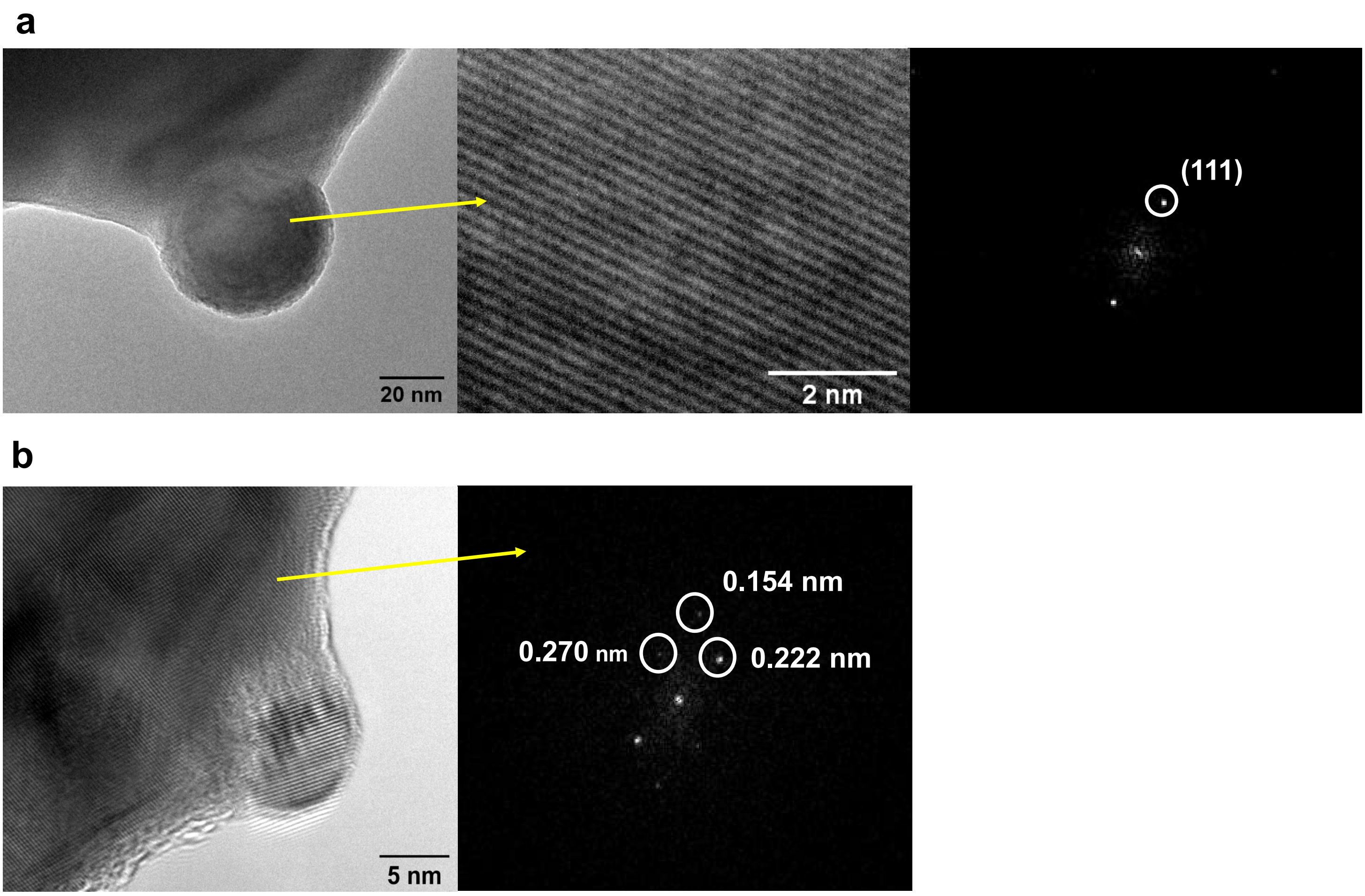

Figure S7. TEM micrographs and Digital Diffraction Pattern (DDP) of the exsolved $La_{0.43}Ca_{0.37}Ni_{0.06}Ti_{0.94}O_{3-\delta}$ after 5 MW cycles. Socketed nature of the NPs and plane (111) of metallic Ni identified, with a measured d-spacing of 0.210 nm **(a)**. Interplanar distances of the parental metallic oxide have also been measured **(b)**, as can be seen in the DDP image. These d-spacings are consistent with perovskite oxides.

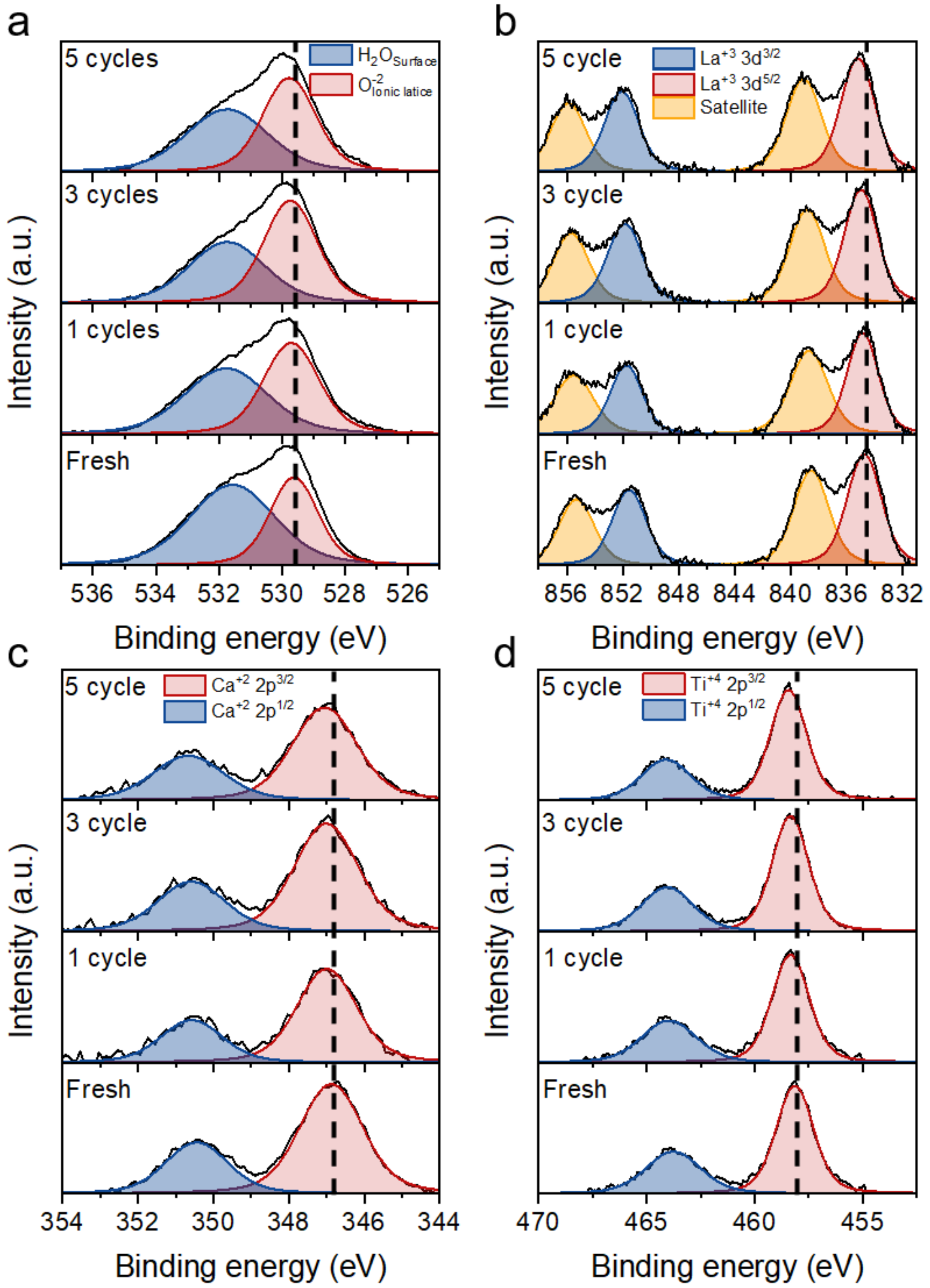

Figure S8. The XPS spectra of O, La, Ca, and Ti (**a**, **b**, **c**, and **d**, respectively) in the $La_{0.43}Ca_{0.37}Ni_{0.06}Ti_{0.94}O_{3-\delta}$ material were obtained before and after MW reduction treatments, which involved various cycle number procedures.

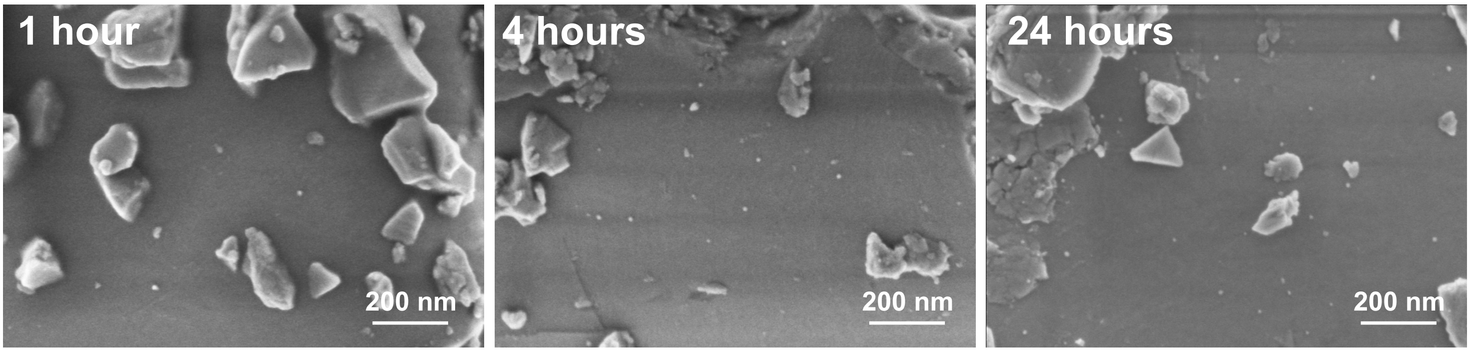

Figure S9. HRFESEM micrographs of LCTN after thermal exsolution at 400 ºC for 1, 4 and 24 hours (5% H_2/Ar flow). These tests reveal remarkable differences between MW and thermal exsolution, especially with respect to the size of the exsolved nanoparticles. Even after 24 h treatment, exsolved NPs are around 10 nm of mean size, way smaller than the MW exsolved ones. This fact states that the reached temperature during MW tests is not the explanation for the growth of the exsolved NPs, and this behavior can be associated with the MW radiation effect. On the other hand, extending the time of the thermal exsolution increases the number of exsolved NPs, as can be seen in these micrographs.

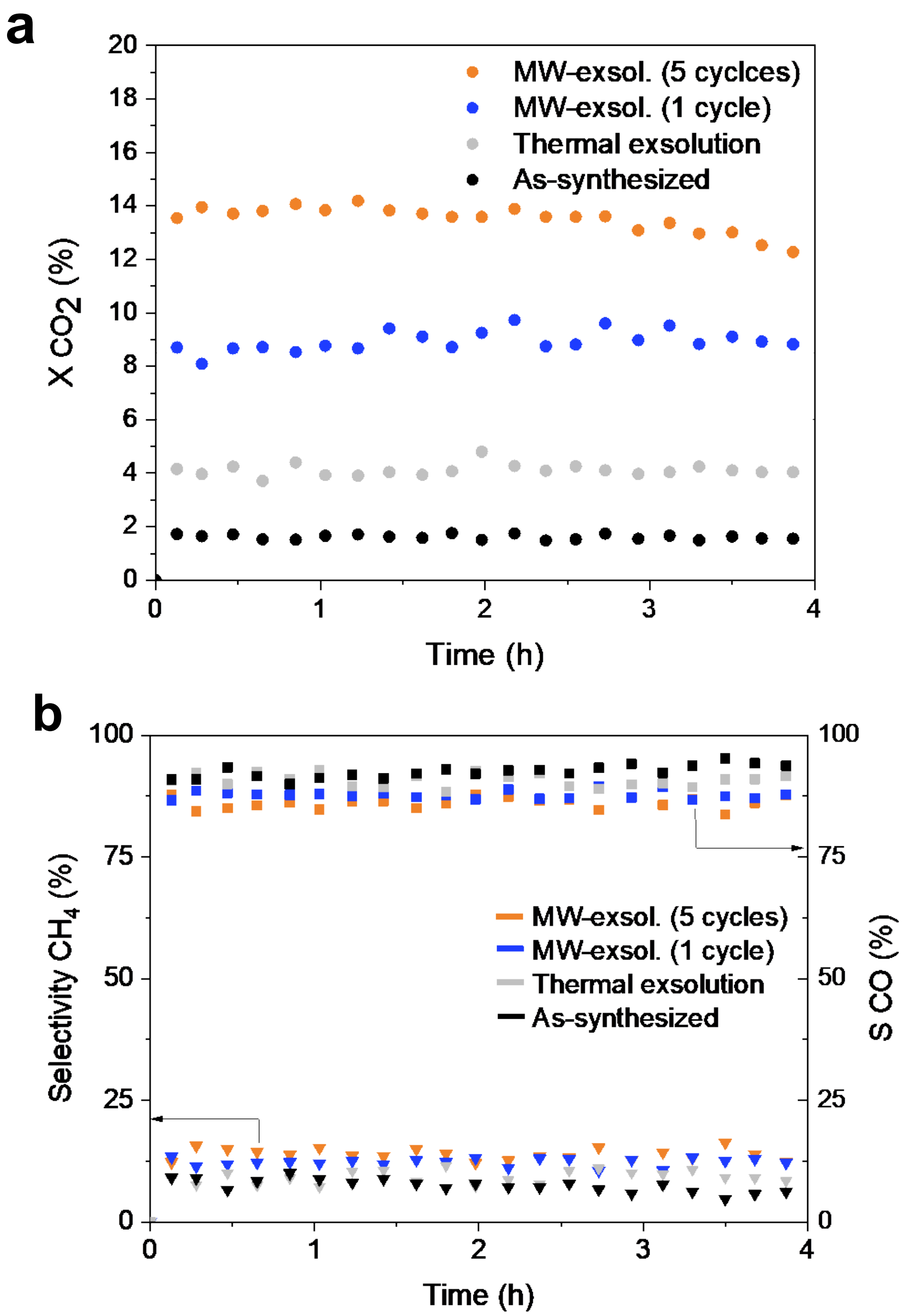

Figure S10. CO_2 conversion **(a)** and selectivity studies **(b)** for $La_{0.43}Ca_{0.37}Ni_{0.06}Ti_{0.94}O_{3-\delta}$ before and after MW reduction treatments, namely 1 and 5 exsolution cycles. Those two treatments were also compared to an in situ thermal exsolution (400 ºC, 1 h under 5% H_2/Ar flow) and with non-exsolved LCTN. All tests were carried out at 400°C and GHSV = 13971 h^{-1}.

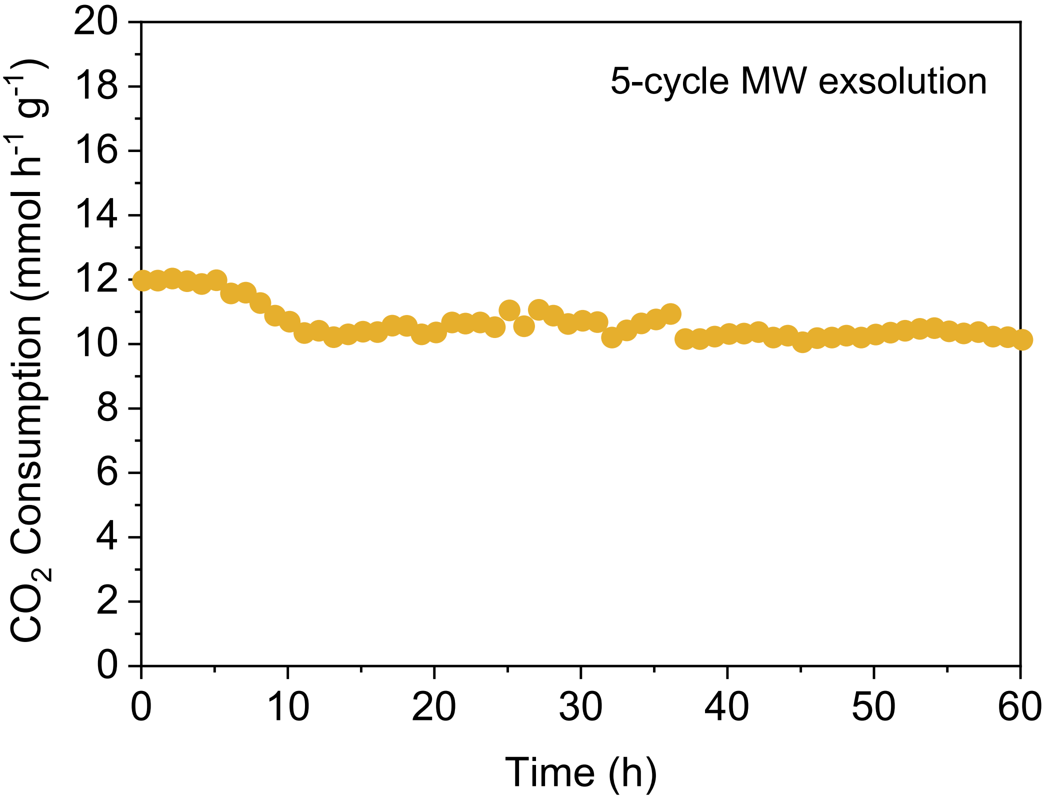

Figure S11. CO_2 hydrogenation tests performed with LCTN after 5 cycles MW exsolution for 60 hours. After an initial slight consumption in the first 10 hours, notable stability was reached during the rest of the experiment.

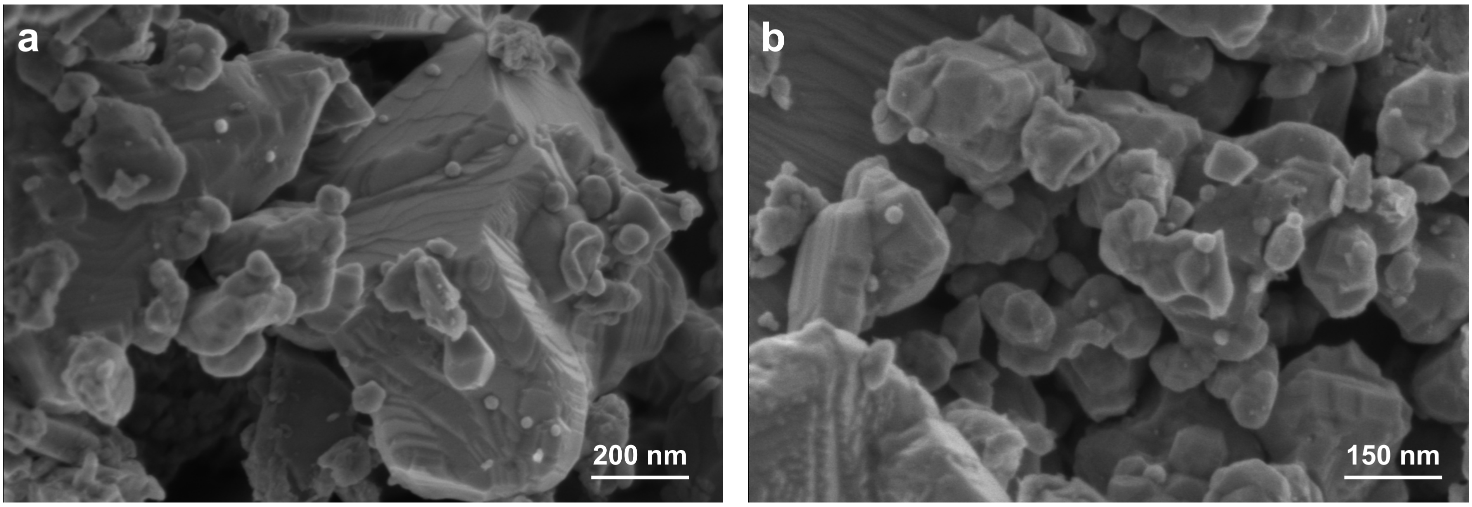

Figure S12. Post-mortem HRFESEM micrographs of exsolved LCTN (5 MW cycles) after **(a)** 4 hours reaction and **(b)** 60 hours tests. MW exsolved NPs remain along the surface in both cases, but some new exsolved smaller NPs can be appreciated, especially after 60 h process. This fact suggests that thermal exsolution is happening during the reaction, due to the presence of H_2 flow.
